# Understanding the Mechanisms That Drive Phage Resistance in Staphylococci to Prevent Phage Therapy Failure

**DOI:** 10.3390/v14051061

**Published:** 2022-05-16

**Authors:** Andrea Jurado, Lucía Fernández, Ana Rodríguez, Pilar García

**Affiliations:** 1 Instituto de Productos Lácteos de Asturias (IPLA-CSIC), Paseo Río Linares s/n, 33300 Villaviciosa, Asturias, Spain; andrea98jurado@yahoo.es (A.J.); anarguez@ipla.csic.es (A.R.); pgarcia@ipla.csic.es (P.G.); 2 DairySafe Group, Instituto de Investigación Sanitaria del Principado de Asturias (ISPA), 33011 Oviedo, Asturias, Spain

**Keywords:** *Staphylococcus*, phage resistance mechanisms, adaptive resistance, phage cocktails

## Abstract

Despite occurring at the microscopic scale, the armed race between phages and their bacterial hosts involves multiple mechanisms, some of which are just starting to be understood. On the one hand, bacteria have evolved strategies that can stop the viral infection at different stages (adsorption, DNA injection and replication, biosynthesis and assembly of the viral progeny and/or release of the newly formed virions); on the other, phages have gradually evolved counterattack strategies that allow them to continue infecting their prey. This co-evolutionary process has played a major role in the development of microbial populations in both natural and man-made environments. Notably, understanding the parameters of this microscopic war will be paramount to fully benefit from the application of phage therapy against dangerous, antibiotic-resistant human pathogens. This review gathers the current knowledge regarding the mechanisms of phage resistance in the *Staphylococcus* genus, which includes *Staphylococcus aureus*, one of the most concerning microorganisms in terms of antibiotic resistance acquisition. Some of these strategies involve permanent changes to the bacterial cell via mutations, while others are transient, adaptive changes whose expression depends on certain environmental cues or the growth phase. Finally, we discuss the most plausible strategies to limit the impact of phage resistance on therapy, with a special emphasis on the importance of a rational design of phage cocktails in order to thwart therapeutic failure.

## 1. Introduction

We are increasingly aware of the significant role that bacteriophages play in the evolution and equilibrium of bacterial populations. Therefore, a greater insight into the interactions between phages and their hosts will help us understand how environmental microbial communities evolve, including those that constitute the human microbiota. Beyond that, a deeper comprehension of the interplay between bacteria and their viruses will be paramount if phages are to be used as antimicrobials so that we can foresee and, hopefully, avoid the past mistakes made with antibiotics. Indeed, phage therapy is considered one of the possible strategies that can help to control the antibiotic resistance crisis, which has become one of the biggest threats to global health.

The rapid acquisition of antibiotic resistance in members of the genus *Staphylococcus*, especially the species *Staphylococcus aureus*, has become a major problem in the clinic. Despite being a commensal of humans and other animals, *S. aureus* is also an important opportunistic pathogen, causing bloodstream and soft tissue infections, ventilator-associated pneumonia, and food poisoning, amongst other affections. Since 1960, different methicillin-resistant *S. aureus* (MRSA) strains have been isolated around the world. These clones are the result of the acquisition of the staphylococcal cassette chromosome *mec* (SCC*mec*), which encodes proteins that confer resistance to β-lactam antibiotics, including methicillin [1]. As an alternative to methicillin, vancomycin has become the treatment of choice against MRSA infections. However, the use of this antibiotic has led to the emergence of vancomycin-resistant *S. aureus* (VRSA). The World Health Organization (WHO) recently released a list of the most dangerous antibiotic-resistant bacteria, in which MRSA and VRSA strains were classified as high priority [2].

In this context, bacteriophages infecting *Staphylococcus* have become the focus of researchers worldwide as a potential treatment against multi-resistant strains [3,4]. However, it is also important to determine the participation of phages in the spread of resistance markers within this genus. According to the currently available data, bacteriophages from three different families within the order Caudovirales (tailed phages) are known to infect staphylococci. These families are *Siphovoridae*, *Myoviridae* and *Podoviridae* (Table 1). Generally, phages belonging to the *Myoviridae* and *Podoviridae* families are virulent, while siphoviruses are temperate bacteriophages. From a therapeutic point of view, virulent bacteriophages are the best option, as they ensure the death of the infected bacteria, since they only carry out the lytic cycle. Moreover, temperate phages might contribute to resistance gene transfer by transduction, and/or alter the expression of virulence factors in the host bacterium. Nevertheless, some strategies have been developed to allow the therapeutic use of temperate phages. These approaches consist in the deletion or suppression of genes required for lysogeny [5].

Each bacteriophage typically has a range of susceptible bacterial hosts that may be as small as one or a few strains within a species, or as large as bacteria belonging to different genera, although the latter possibility is very rare. The ability to predict this range would facilitate the rational design of specific phage mixtures (cocktails) for the treatment of bacterial infections, both in clinical and industrial settings, with a high guarantee of success. In this context, it is worth noting that virulent phages infecting *Staphylococcus*, belonging to the families *Myoviridae* and *Podoviridae*, tend to exhibit a wide host range, most notably myophages. Due to natural evolutionary processes, bacteria have developed resistance mechanisms to survive viral infection, thus reducing the range of susceptibility to a given phage and, therefore, its therapeutic potential. To overcome this drawback, it is necessary to have detailed knowledge of the mechanisms by which *Staphylococcus* phages infect their host cells and how bacterial cells develop resistance to these viruses. On the other hand, it is equally important to understand the evolutionary strategies by which phages overcome bacterial resistance development. This review aims to summarize all of the currently available information in this regard, including not only mutational changes that lead to phage resistance, but also adaptations that may temporarily increase the ability of a bacterial population to withstand viral infection. This phenomenon is associated with specific environments and, consequently, difficult to detect in the laboratory, but it might have a significant impact on therapeutic success. Additionally, phage counter-defensive strategies against the different resistance mechanisms will be discussed.

## 2. Phage Resistance Determinants

**Figure 1:**
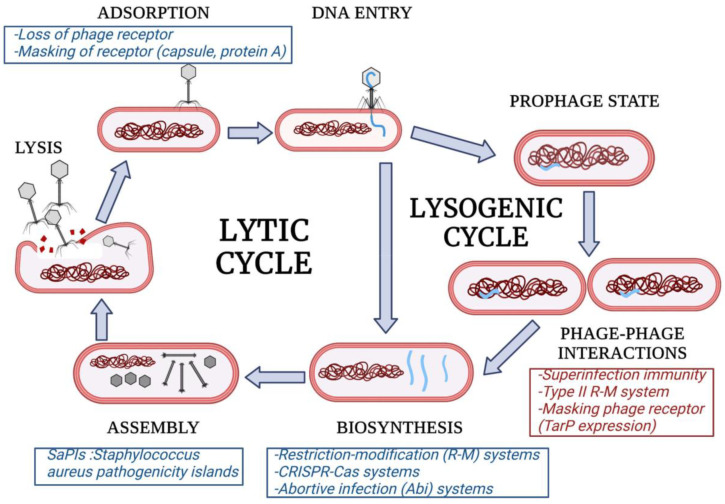
Summary of phage resistance determinants interfering with different stages of the lytic life cycle (blue boxes) and resistance mechanisms encoded in prophages (red boxes).

The life cycle of a virulent phage consists of the following stages: adsorption, where receptor-binding domains (RBDs) bind specifically to the bacterial surface; injection of genetic material, viral DNA or RNA is introduced through the tail of the phage into the bacterium; replication of viral DNA (lytic cycle) or integration of viral DNA into the bacterial genome (lysogenic cycle); transcription and translation of viral protein structures (in the lytic cycle from the beginning and in the lysogenic cycle once the prophage is activated); assembly of the new viral particles; and bacterial lysis, which allows the release of the phage progeny to the outside (Figure 1). In order to withstand phage infections, bacteria have acquired mechanisms that may interfere with one of these phases, leading to decreased susceptibility or even resistance. Below, we will describe the currently available information concerning these mechanisms in staphylophages. If known, phage counter-defensive strategies against such mechanisms will also be indicated.

### 2.1. Inhibition of Phage Adsorption

Wall teichoic acid (WTA), a major structural component of the cell wall of Gram-positive bacteria, acts as a receptor for most *Staphylococcus* phages and, consequently, plays a crucial role in the phage adsorption step. In fact, only one phage (ΦSLT) has been shown to bind to lipoteichoic acid, also present in the bacterial wall, and use it as a receptor [6]. The main structure of WTA in this genus is a backbone of 1,5-ribitol-phosphate (RboP) molecules to which D-alanine molecules and α-GlcNAc and/or β-GlcNAc residues are added by the products of the *dlt* operon and transferases TarM and TarS, respectively. However, the WTA of some *S. aureus* strains belonging to lineage ST395, CoNS, *Staphylococcus pseudintermedius* and *Staphylococcus carnosus* consists of 1,3-glycerol-phosphate (GroP) molecules decorated with D-alanine and α-GalNAc molecules (added by the TarN transferase enzyme and its homologs) [7].

**Figure 2:**
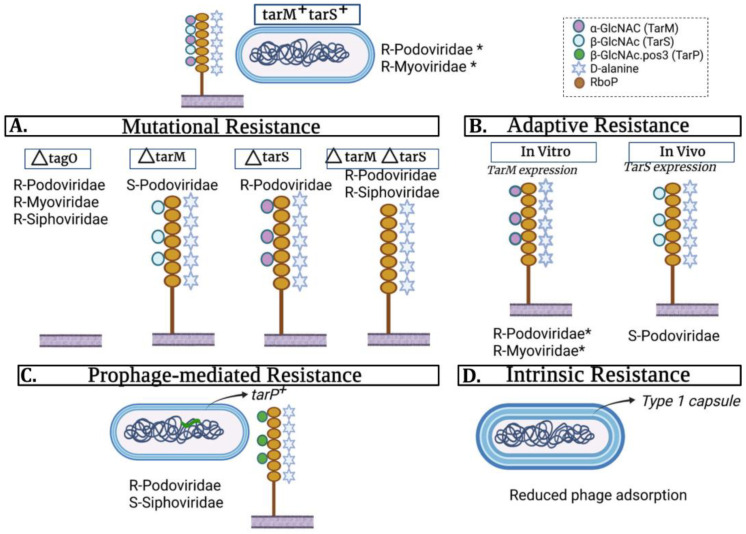
Different types of phage resistance mechanisms affecting WTA structure. The drawing on top represents a hypothetical wild-type strain, which may then display differences in its susceptibility to different phages due to mutational resistance (**A**), adaptive resistance (**B**) or prophage-mediated resistance (**C**). Additionally, some strains may exhibit reduced adsorption of all types of phages if they have a type 1 or 2 capsule (**D**). R-: resistance to a family of phages, S-: susceptibility to a family of phages. *, α-GlcNAc can mask the WTA backbone and/or β-GlcNAc, which has an impact on the attachment of certain bacteriophages. The box in the top right is the legend for the different symbols of WTA components and modifications.

The recognition of this receptor has different specificities depending on the bacteriophage family (Table 1). For instance, bacteriophages of the *Myoviridae* family adsorb to the WTA backbone [8] and, in some cases, also α-GlcNAc [9] or β-GlcNAc [10]. This occurs thanks to the existence of multiple receptor binding proteins (RBPs) in some myoviruses, a trait that often results in a broad host range [9]. In turn, bacteriophages of the *Podoviridae* family bind WTA consisting of RboP molecules bound to β-GlcNAc. Finally, bacteriophages of the *Siphoviridae* family bind to WTA decorated with α-GlcNAc and/or β-GlcNAc [8]. Within this family, phage Φ187 has the ability to infect bacteria that have WTA consisting of GroP molecules bound to α-GalNAc due to the possession of TagN (α-O-GalNAc transferase), TagV (nucleotide sugar epimerase) and TagF (short GroP WTA polymerase) genes [7]. From this, it can be gathered that the structure of WTA in a given strain will be decisive regarding the infective capacity of different bacteriophages (Figure 2A).

Deletions or point mutations in the undecaprenyl-phosphate *N*-acetylglucosaminyl 1-phosphate transferase *tagO* gene (necessary in the early stages of WTA formation) confer resistance to phages belonging to the three families, including *Myoviridae* (Φ812, ΦK, ΦSA012, phiIPLA-RODI) [8,10,11]. However, some myophages, like ΦSA039, require the presence of both TagO and TarS activity to infect the host cells [10]. Interestingly, infection of *S. pseudintermedius* by this same phage is prevented by WTA glycosylation [12]. In the case of the *Podoviridae* family, resistance may occur as a result of the deletion or inactivation of the *tarS* gene or the simultaneous activity of the *tarS* and *tarM* genes [13]. Indeed, decoration of WTA with α-GlcNAc masks β-GlcNAc, the receptor of podoviruses, impeding their adsorption. An exception to this is phage ΦS24-1, which is able to attach to WTA glycosylated by either β-GlcNAc or α-GlcNAc, although it cannot infect coagulase-negative staphylococci (CoNS), so its infection range is not as broad as that of *Myoviridae* phages [14]. Finally, resistance to phages of the *Siphoviridae* family may be caused by insertions in the *tarM* gene, as shown for phages ΦSa2mW, Φ47, ΦP13 and Φ77 [8]. Additionally, Winstel et al. [7] observed that *tagN* mutants derived from *S. aureus* ST395, a strain that produces GroP-WTA decorated with α-GalNAc, were resistant to phage Φ187.

In response to receptor changes in the host population, bacteriophages also undergo mutational changes in the context of co-evolutionary dynamics. For example, a study reported that phage ΦSA039 accumulated mutations in open reading frames (ORFs) 100 and 102 that allowed adsorption to a resistant mutant of its host strain lacking the β-GlcNAc residue bound to the WTA [12].

In addition to changes in the structure of WTA, adsorption may also be impacted by mutations affecting the production of extracellular factors such as protein A or the capsule [15,16]. However, these changes are often transient and respond to variations in the environmental conditions. As such, this phenomenon will be discussed later in this review (Section 3). Finally, Azam et al. [10] identified other genes whose mutations reduced phage adsorption, although their precise role is still to be determined. These include *gmk* (guanylate kinase), *yozB* (putative membrane protein), *murA2* (the gene for UDP-N-acetylglucosamine 1-carboxyvinyltransferase), *rapZ* (RNase adapter protein), *rpoA* (DNA-dependent RNA polymerase) and *scd* (iron–sulfur repair protein).

### 2.2. Interference with Phage Biosynthesis

#### 2.2.1. Restriction–Modification (R-M) Systems

The best characterized bacterial strategy to eliminate foreign DNA, such as plasmids and phages, is restriction–modification (R-M). R-M systems have been identified in 90% of prokaryotes, and consist of two enzymatic activities (endonuclease and methyltransferase) that target the same short nucleotide sequence [17,18]. These systems consist of a set of genes that code for proteins with complementary activities. One gene (*hsdR* or *res*) codes for a restriction endonuclease that breaks unmodified DNA, and another gene (*hsdM* or *mod*) encodes a DNA adenine or cytosine methyltransferase that modifies host DNA to avoid the action of endonucleases. Additionally, in the case of bacteria with type I R-M systems, there is a third gene (*hsdS*) whose product is a DNA-binding protein that signals target sequences for breakage or modification. There are four currently known R-M systems (I, II, III, IV) in *Staphylococcus* that differ in the active sites of these enzymes and in the complexes formed between them [19]. In *S. aureus*, the most abundant system is type I, with Sau1 being the most common [20]. The target recognition domains (TRD) of the HsdS subunit of Sau1 are the lineage- and clonal complex (CC)-specific sequences that signal the location of the active site to the rest of the enzymes in the system [20,21]. Most of the sequences of the TRDs found in Sau1-positive *S. aureus* strains are currently available. As a result, sequencing the genome of candidate phages for therapeutic applications would help to define a range of susceptibility to phages by CC [22]. This same idea could be extrapolated to another system present in *Staphylococcus*: the type IV system represented by the SauUSI gene [23]. In contrast, type II systems usually encoded in phages and mobile genetic elements only show specificity for certain strains depending on their sequence type, and are not conserved within a CC [17]. Therefore, they are not as good a determinant of phage susceptibility range as type I systems.

The efficacy of R-M systems depends on the relative activity of the endonuclease and the methylase enzymes. Usually, the nuclease has a faster processing rate, resulting in degradation of the viral DNA. Nonetheless, it must be highlighted that if the phage genome becomes methylated, it will escape degradation by the restriction enzyme and the infection will proceed. Furthermore, the resulting virions will be able to propagate in other bacterial cells with the same methylation pattern. Additionally, some phages have evolved genome sequences lacking the restriction sites recognized by these enzymes. The best-known example of this is phage K, which does not have any *Sau*3A recognition sites [24]. Another potential strategy is the acquisition of methylase genes by the virus, as has been already observed in phages infecting *Lactococcus lactis* [25]. This phenomenon has not been found so far in staphylophages.

#### 2.2.2. CRISPR-Cas Systems

Besides R-M systems, bacteria also possess an additional mechanism to degrade invading genetic material, but in this case, the endonuclease recognition sequences are directly derived from the virus. Clustered regularly interspaced short palindromic repeats (CRISPRs) are a defensive strategy in which bacteria infected by a virus incorporate fragments of viral DNA into spacer regions of their own genetic material [26]. These viral DNA fragments then work as an acquired immunization of the bacterium against new infections by the same virus. Notably, when the cell divides, this immunological memory will be passed on to its offspring. CRISPR-associated genes (Cas) encode the enzymes responsible for processing the CRISPR-RNA precursor transcript into crCRIPSR-RNA transcripts that can bind to other Cas enzymes to form effector complexes. These complexes will specifically recognize complementary sequences (corresponding to the phage genome) and cause its destruction by the action of Cas enzymes.

All research so far indicates that CRISPR-Cas systems are not very widespread in the genus *Staphylococcus* compared to other bacteria [27], even though several studies have searched for these systems in the genomes of staphylococci, especially *S. aureus*. For instance, a recent study that analyzed 716 whole genomes available in public databases found that only 0.83% carried CRISPR-Cas systems, all of which belonged to subtype IIIA [28]. A similar percentage had already been observed by Cao et al. [29] after probing the genomes of 616 clinical isolates. Other studies encountered higher prevalence of these sequences, such as the one conducted by Wang et al. [30], which identified CRISPR loci in 12.92% of the analyzed strains. CRISPR-Cas systems have also been detected in species other than *S. aureus*, such as *S. epidermidis*, *Staphylococcus pseudointermedius*, *Staphylococcus haemolyticus*, *Staphylococcus cohni* and *Staphylococcus lugdunensis* [31,32,33].

The most commonly found subtypes in *S. aureus* are IIA and III-A [30]. Interestingly, the latter tends to be located in or near the SCC*mec* region, which suggests its potential mobilization by HGT, as was recently demonstrated by Watson et al. [34]. On top of that, an article by Mo et al. [35] found that immunity mediated by type IIIA systems can increase the mutation rate through non-specific DNAse activity. This finding has important implications from the perspective of resistance development both to antibiotics and bacteriophages.

In order to counteract the activity of CRISPRs, bacteriophages can also encode small proteins called anti-CRISPRs (Acrs) that block the antiviral immunity provided by these systems, thereby allowing phage infection to proceed [36]. In *S. aureus*, Watters et al. [37] identified several Acrs (AcrIIA13, AcrIIA14 and AcrIIA15) that inhibit Cas9 activity. Data from other microorganisms seem to indicate that these proteins are most commonly found in temperate rather than virulent phages [38,39], but only the latter are generally used in phage therapy. Nonetheless, the presence of Acrs in a phage genome would be an interesting asset regarding its therapeutic potential. On the other hand, identification of potential spacer sequences (SSc) in the genome of a target strain would help predict which phages are the most adequate for treatment. For instance, a recent study found spacer sequences (SSc) in the genome of *S. aureus* strains from various sources that matched sequences corresponding to phages that had previously been considered as a treatment against this pathogen [28].

#### 2.2.3. Abortive Infection

Abortive infection (Abi) systems can inhibit phage propagation by targeting DNA replication, transcription or translation, and ultimately lead to the death of the infected cell [40]. It would be a “sacrifice” for the survival of the community. An Abi system, Stk2, has been identified in *S. aureus* and *S. epidermidis*. Stk2 becomes activated upon infection by certain *Siphoviridae* phages, and phosphorylates multiple bacterial proteins, which results in cell death [41]. A recent study describing the high throughput search for phage resistance determinants in *S. aureus* genomes available in databases reported the identification of the *abiR* gene in only 13 strains [40]. This same work found the presence of retrons (another Abi system based on a reverse transcriptase (RT) and a non-coding RNA (ncRNA)) in the genomes of many staphylococcal strains.

Abi systems are generally activated by the presence of certain phage proteins or peptides. As a result, phage variants with mutations in these proteins do not trigger the death of the infected cell and are able to escape abortive infection [42]. This phenomenon has not been observed in phages infecting *Staphylococcus* yet, but there are examples in viruses infecting other microorganisms, like *Escherichia coli*.

### 2.3. Assembly Interference

Staphylococcal pathogenicity islands (SaPIs) are phage-related genomic elements that frequently encode virulence factors and/or antibiotic resistance determinants [43]. In order to spread between bacteria, these islands need to parasitize a temperate phage called a helper. The life cycle of the SaPI starts when the helper phage infects the host cell or when a resident prophage is activated by the SOS response. Then, a viral protein derepresses the SaPI repressor (Stl), leading to proliferation of the chromosomal island. In some cases, the regulatory cascade triggered by the derepressor protein encoded by the helper phage is far more complex and involves the sequential activation of several SaPIs [44]. This derepressor function can be played by different proteins, such as dUTPases, the Sri protein, the product of ORF15 from phage 80α or DUF3113 [45]. Later on in the process, certain proteins encoded in the SaPI interfere with phage assembly. These include the phage packaging interference (Ppi) proteins, which prevent the activity of the phage terminase and favor packaging of the SaPIs in the phage capsids [46]. Upon release, the newly formed particles will disseminate the SaPIs to new host cells, inside which they will integrate into the chromosome, and will not lead to cell lysis. However, siphoviruses can acquire resistance to SaPIs, for instance, through mutations affecting the derepressor proteins [47].

In practical terms, this phenomenon would only affect the use of virulent mutants derived from temperate phages, in which case it would be important to determine whether they can act as helpers for SaPI mobilization. If that is the case, they would not be good candidates for phage therapy unless the gene coding for the derepressor has also been removed from the viral genome.

## 3. Environment-Driven Adaptations That Enhance Phage Resistance

Most research on phage resistance focuses on the acquisition of DNA fragments or mutations that confer the ability of previously susceptible strains to withstand viral predation in a stable manner. Obviously, such changes are very important, especially in the context of phage therapy. Nonetheless, temporary adaptations of bacterial cells to a specific environment may also be associated with variations in phage susceptibility. These adaptive changes are currently much less known. However, attaining a better understanding of their impact will be significant, not only from the perspective of characterizing phage–host interactions in nature, but also to predict and avoid possible therapeutic failure.

In some bacterial species, there is evidence that differential expression of resistance determinants may modulate susceptibility in different environments or growth phases. For instance, quorum sensing regulates the activity of CRISPR-Cas systems in *Serratia* [48] and *Pseudomonas aeruginosa* [49]. In turn, quorum sensing downregulates the expression of a viral receptor porin (OmpK) in *Vibrio anguillarum* [50]. Autoinducers also downregulate phage receptors in *Vibrio cholerae* [51].

In *Staphylococcus*, information in this regard remains scarce, but a few recent studies are shedding some light. For instance, it is now known that WTA glycosylation in *S. aureus* is regulated by environmental factors [52]. Indeed, a recent study reported that the expression levels of *tarM* and *tarS* differed between in vitro conditions (α-GlcNAc WTA) and during the infection (β-GlcNAc WTA), a result that bears relevance on the efficacy of certain phages as therapeutics (Figure 2B). Additionally, Moller et al. [53] observed that the deletion of *phoR* heightened phage susceptibility. Interestingly, it is known that the two-component system PhoRS promotes the degradation of glycerol phosphate WTA in *S. aureus* and *B. subtilis* as a phosphate-scavenging strategy [54,55]. In *B. subtilis*, this response also involves the downregulation of WTA biosynthesis, but it is not known if this is also the case in *S. aureus* [56]. Based on this, the authors hypothesize that phosphate starvation may lead to PhoR-regulated phage resistance. The same study found other interesting susceptibility determinants that might give us clues about the potential effects of different environmental conditions on the ability of *S. aureus* populations to fight phage infection. One of them is the homolog of *relA* in this bacterium, which is frequently referred to as *rsh*. A mutant in this gene exhibited increased susceptibility to phages, although complementation did not restore the wild-type phenotype. It is worth noting that a previous study found that biofilms developed under low-level phage predation exhibited upregulation of this gene [57]. Indeed, subsequent work demonstrated that this dysregulation pattern was observed in several staphylococcal strains, coinciding with the production of DNA-rich biofilms upon phage infection [58]. Moreover, a mutant lacking the synthase domain of RSH displayed a lesser increase in biofilm formation when phage phiIPLA-RODI was present, indicating that the stringent response might be involved in this phenomenon. Although not a bacterial adaptation, this article also shows how an environmental factor, more specifically pH, can modulate the interplay between phage and host by inactivating part of the viral population, and as a result enable phage–bacteria co-survival.

Biofilm-forming bacteria generally exhibit increased resistance to antimicrobials due, to some extent, to the presence of an extracellular matrix that hinders their access to the cells [59]. However, this does not seem to be the case for phages infecting the genus *Staphylococcus*. Indeed, all evidence so far suggests that bacteriophages can penetrate the biofilm and infect susceptible sessile cells [60], even in the context of mixed-species biofilms [61,62,63,64]. Cerca et al. [65] showed that *S. epidermidis* biofilms were as susceptible to phage K as stationary-phase cultures. It must also be noted that some phages infecting *Staphylococcaceae* produce polysaccharide depolymerases that may also help degrade the extracellular matrix and/or capsular polysaccharides [66]. Capsule production is thought to play a role in making phage adsorption more difficult in this genus, at least capsule types 1 and 2 [16] (Figure 2D). For the most abundant capsule types (5 and 8), this correlation may not be so clear, but it remains a possibility. Therefore, the biosynthesis of phage enzymes able to degrade these bacterial surface structures would be a significant advantage for the virus. Finally, the accumulation of another component of *S. aureus* biofilms, protein A, can also limit access of the phages to the cell surface [15]. The expression of *spa*, the gene coding for protein A, is under the control of many regulators, including Agr, SarA, SarS and Rot, amongst others [67]. This protein is generally expressed during the exponential phase of growth and is subsequently downregulated, which might have an effect on viral attachment.

Overall, it is becoming increasingly clear that temporary changes in the surrounding milieu may affect how bacterial cells respond to phage infection. As a result, understanding these variations will be important to determine the potential of certain phages to succeed in eliminating an infection under certain environmental conditions.

## 4. Phage–Phage Interactions

In addition to host-encoded determinants, phage resistance can be due to the impact of other phages, especially prophages, competing for taking full control of the infected cell. Given that most staphylococcal strains are lysogenic, such mechanisms would be expected to play a major role in the result of a viral attack.

One such mechanism is superinfection immunity, which requires the presence of temperate phages in the bacterial chromosome encoding homologs of the cI repressor, which would block the lytic cycle of other infecting phages with binding sites for this protein [68]. A recent study by Moller et al. [40] suggests that superinfection immunity correlates with empirical resistance to temperate phages. In the context of phage therapy, it would be necessary to avoid using phages belonging to the same superinfection group as prophages present in the target staphylococcal strain. Otherwise, the applied treatment would not succeed in eliminating the infection.

Superinfection exclusion (Sie) systems prevent the entry of the viral DNA into the bacterial cells, and are mediated by proteins often encoded in prophages. These systems have been identified in Gram-positive bacteria, like *Lactococcus lactis*, but there is no information regarding their existence in staphylococci yet [69].

Prophages may sometimes contribute to defending their host cell from superinfecting phages by promoting the cleavage of the invading phage genome. This occurs through the action of type II R-M systems, which can sometimes be harbored in prophages, as is the case of Sau42I [70].

Some temperate phages can also harbor genes leading to modifications of the bacterial surface that alter recognition by other bacteriophages. An example of this is the *tarP* gene, encoded by prophages present in MRSA strains belonging to the clonal complexes CC5 and CC398. The product of this gene is an enzyme responsible for adding a β-GlcNAc residue at position 3 of the WTA (instead of position 4, as happens due to the activity of TarS) [71]. This change enhances susceptibility to siphoviruses, while making the cells resistant to podoviruses. Therefore, if phages from the family *Podoviridae* are being considered for treatment, it will be important to establish whether TarP is present in the target bacterial strain (Figure 2C).

## 5. Overcoming Phage Resistance in Therapeutic and Biocontrol Applications against *S. aureus*

As has been observed for other antimicrobials, the development of phage resistance during treatment may lead to therapeutic failure. The solution to this problem partly lies in the incredible diversity and abundance of phages in nature, which provides an almost infinite source of new viruses able to infect the strains that acquired resistance to the previously utilized phages. However, in order to fully take advantage of this, it is essential to determine the best plan of action. With this purpose in mind, the most widespread strategy aimed at increasing the likelihood of phage therapy success is the use of mixtures of different viruses called phage cocktails. In addition to making resistance more difficult to develop, these preparations increase the phages’ ability to evade the immune system [72].

**Figure 3:**
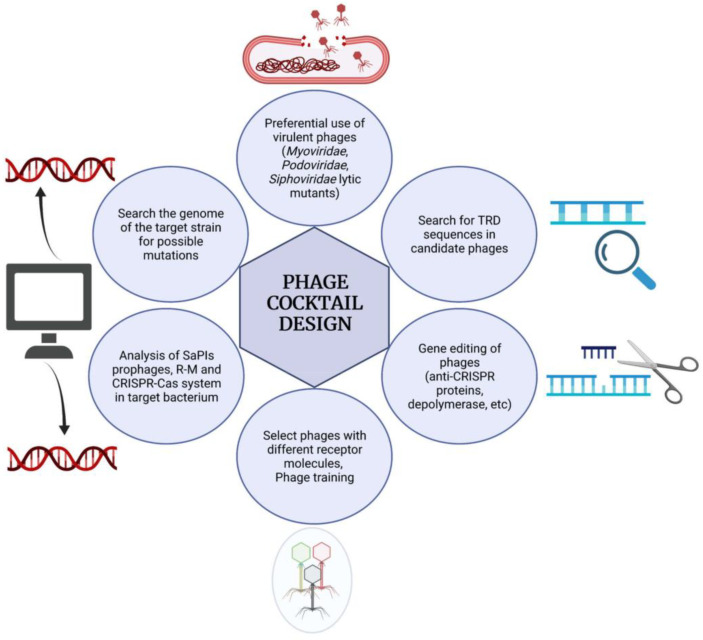
Schematic representation of different aspects that must be considered for rational phage cocktail design.

The success of these combinations relies on the fact that if some bacterial cells acquire resistance to one phage in the cocktail, they will remain susceptible to other phages in the mixture. The frequency of multi-resistance acquisition is usually quite low and, as a result, treatment would be able to eliminate the infection. Nevertheless, it must be emphasized that for this to work properly, it is essential to carefully choose the right phage combination. Most phage cocktails designed against staphylococcal infections exclusively include phages belonging to the *Myoviridae* family [72,73,74]. The main reason for this is their broad host range compared to the other two families (*Siphoviridae* and *Podoviridae*) coupled with their lytic life cycle that ensures bacterial death, and the presence of fewer virulence genes compared to lysogenic phages. However, there are some exceptions to this rule in which virulent phages from the *Podoviridae* family are also included. One example of this is a product (Pyophage) marketed by Microgen (Russia), which consists of a myovirus (vB_SauM_fRuSau) and a podovirus (SCH1). This product was effective against 97% out of 31 MRSA strains and 85% out of 20 MSSA strains [75]. The lytic range of this cocktail was further increased in a subsequent work by selecting new phage variants derived from the original cocktail [76]. The application of members of the *Siphoviridae* family is more limited due to their temperate lifestyle, although virulent mutants can be obtained and then used in therapy [77]. This would allow a greater range of options, especially in terms of bacterial receptors. Nevertheless, as has been mentioned throughout the text, it is important to first establish whether the target strain carries resistance determinants that may prevent infection by the candidate siphovirus. This would include, for instance, analysis of the SaPIs or prophages present in the bacterial genome. For instance, presence of gene *tarP*, which is common in MRSA strains, indicates that podoviruses are not a good therapeutic option. In contrast, strains with truncated *tarM*, which is relatively frequent in mastitic cow isolates, are susceptible to phages from the *Podoviridae* family [72]. Genome analysis of the bacterial strain to be eliminated would actually be beneficial for the selection of all types of phages, as it would enable the identification of other resistance determinants, such as R-M or CRISPR-Cas systems. In that sense, recent high throughput studies are providing a large amount of information regarding the genetic factors involved in host-range determination [40,53]. The results obtained in such studies will be paramount to pinpoint the most important genes or genomic regions to be analyzed for the selection of therapeutic phages against a particular isolate and, ultimately, allow rational design of the best cocktails (Figure 3).

An additional strategy to further improve the results of phage therapy and hamper the development of resistance involves taking advantage of our knowledge regarding phage–host coevolution. For instance, the approach known as phage training has been shown to lead to the selection of more efficient phage variants. Borin et al. [78] observed that coevolution of a candidate phage with its *E. coli* host selected trained phages that were able to kill not only the original strain, but also phage-resistant mutants. Moreover, another study revealed that a combination of slightly different phage variants as a strategy to increase genotypic diversity halted phage resistance evolution in the bacterial population [79]. A recent study utilized phage training to isolate improved variants of three wild-type phages against *S. aureus* [80].

Phage diversity may also be increased by genetic engineering, an approach that allows the targeted, specific manipulation of phage genomes to develop new candidate variants [81,82]. The many changes that can be attained with this strategy include the elimination of lysogeny genes from phage genomes, the addition of new RBPs to increase the host range, the deletion of virulence genes from the phage genome and the introduction of a gene that can lower bacterial resistance to antibiotics. So far, this method has not been frequently used in staphylophages. Nevertheless, there is one study in which a *S. aureus* phage was engineered to be a delivery vehicle for an antibacterial protein [83].

Last but not least, phages may be used to complement antibiotic therapy, especially if it is possible to take advantage of the phenomenon known as phage–antibiotic synergy (PAS). For example, the combination of phage SB-1 with rifampin or daptomycin was successful in eliminating MRSA strains not only in the planktonic state, but also in recalcitrant biofilms, whereas the antibiotics alone could only kill planktonic cells [84]. Moreover, pretreatment of the biofilms with the same phage and subsequent treatment with one of five antibiotics (rifampin, daptomycin, fosfomycin, ciprofloxacin or vancomycin) was even more successful than co-treatment, and reduced the number of persister cells. However, this approach needs to be examined for each antibiotic–phage combination to make sure that there is a synergistic interaction between the two and, especially, to avoid potential antagonism [85].

## 6. Conclusions

Phage therapy has been proposed as a viable, safe strategy to treat recalcitrant bacterial infections caused by staphylococci. Although phage resistance development is also possible, bacteriophages offer some advantages that chemotherapy does not have. For instance, phages can actively co-evolve during treatment, and new variants that infect the bacterial-resistant mutants can spontaneously appear. Moreover, there is an endless reservoir of novel candidate phages in nature, which would enable the selection of a new virus if the infection has become resistant to the previous one. The natural repertoire of candidate phages can also be supplemented by the use of strategies such as phage training and the genetic engineering of phages. Nonetheless, in order to select the combination of phages most likely to succeed against a given infection, it remains necessary to acquire as much information as possible about the mechanisms that affect host range and/or confer resistance to different phages. This knowledge will eventually allow the efficient tailor-made design of the most adequate phage cocktails for each infection.

## Figures and Tables

**Table 1:** Summary of the main characteristics of the three phage families that infect staphylococci.

Family	Tail Morphology	Genome Size (kb)	Life Cycle	Receptor Molecule ^1^
*Myoviridae*	Long, non-flexible, contractile	120–140	Virulent	WTA backbone and sometimes α-O-GlcNAc or β-O-GlcNAc
*Siphoviridae*	Long, flexible, non-contractile	39–43	Temperate	α-O-GlcNAc and/or β-O-GlcNAc
*Podoviridae*	Short, non-contractile	120–140	Virulent	β-O-GlcNAc

^1^ GlcNAc: N-acetylglucosamine, WTA: wall teichoic acid.

## Data Availability

Not applicable.
